# Optimising COVID-19 Vaccination Policy to Mitigate SARS-CoV-2 Transmission within Schools in Zimbabwe

**DOI:** 10.3390/vaccines9121481

**Published:** 2021-12-15

**Authors:** Grant Murewanhema, Solomon Mukwenha, Tafadzwa Dzinamarira, Zindoga Mukandavire, Diego Cuadros, Roda Madziva, Innocent Chingombe, Munyaradzi Mapingure, Helena Herrera, Godfrey Musuka

**Affiliations:** 1 Unit of Obstetrics and Gynecology, Faculty of Medicine and Health Sciences, University of Zimbabwe, Harare, Zimbabwe; gmurewanhema@yahoo.com; 2 ICAP at Columbia University, Harare, Zimbabwe; sm4803@cumc.columbia.edu (S.M.); ic2421@cumc.columbia.edu (I.C.); mpm2189@cumc.columbia.edu (M.M.); gm2660@cumc.columbia.edu (G.M.); 3 School of Health Systems & Public Health, University of Pretoria, Pretoria 0002, South Africa; 4 Center for Data Science and Artificial Intelligence, Emirates Aviation University, Dubai P.O. Box 53044, United Arab Emirates; zindoga.mukandavire@emirates.com; 5 Department of Geography and Geographic Information Science, University of Cincinnati, Cincinnati, OH 45221, USA; cuadrodo@ucmail.uc.edu; 6 School of Sociology and Social Policy, University of Nottingham, Nottingham NG7 2RD, UK; roda.madziva@nottingham.ac.uk; 7 School of Pharmacy and Biomedical Sciences, University of Portsmouth, Portsmouth PO1 2UP, UK; helena.herrera@port.ac.uk

**Keywords:** COVID-19, vaccination, schools, Zimbabwe

## Abstract

The COVID-19 pandemic has disrupted the learning of millions of children across the world. Since March 2020 when the first cases of COVID-19 were reported in Zimbabwe, the country, like many others, has gone through periods of closing and re-opening of schools as part of the national COVID-19 control and mitigation measures. Schools promote the social, mental, physical, and moral development of children. With this viewpoint, the authors argue that schools should not be closed to provide a measured and efficient response to the threats posed by the COVID-19 epidemic. Rather, infection prevention and control strategies, including vaccination of learners and teachers, and surveillance in schools should be heightened. The use of multiple prevention strategies discussed in this viewpoint has shown that when outbreaks in school settings are adequately managed, the transmission usually is low. The information presented here suggests that schools should remain open due to the preponderance of evidence indicating the overriding positive impacts of this policy on the health, development, and wellbeing of children.

## 1. Introduction

The COVID-19 pandemic has disrupted the learning of millions of children across the world. The United Nations Educational, Scientific and Cultural Organization (UNESCO) views educational recovery as a critical priority to avoid a generational catastrophe [1]. For developing countries, some of which are still lagging behind with education, especially for girls and women, rapid restoration is critical to avoid losing the gains [2]. Globally, strategies to make the school environment safer regarding SARS-CoV-2 transmission for both students and service providers is critical for pandemic control whilst allowing educational activities to continue with minimal disruptions [3].

Since March 2020 when the first cases of COVID-19 were reported in Zimbabwe, the country, like many others, has gone through periods of closing and re-opening of schools as part of the national COVID-19 control and mitigation measures [4]. During the first two waves between March 2020 and March 2021 [5], schools were wholly or partly closed at different intervals [6]. With the arrival of the third wave in June 2021 [7], schools were fully closed again [8]. This last wave began to settle by the end of July 2021, and by the end of August, incident cases and fatalities had plummeted significantly. Following protracted periods of school interruptions, it became necessary for the government to re-open schools, prioritising grades sitting state examinations and eventually opening for all students [6]. Schools promote the social, mental, physical, and moral development of children. Therefore, concerns were raised regarding the protracted closure of schools, when the burden of COVID-19 among school-going children and the associated morbidity and mortality in this population has generally, although not universally, been low globally [9]. Some concerns identified of heightened public health significance potentially linking with school closures resulting from the COVID-19 pandemic include an increase in the incidence of unwanted teenage pregnancies, child marriage, illicit substance use, and child labour [10,11]. Similar concerns have been reported elsewhere in Uganda, where it is estimated that nearly a third of the country’s students may never return to school, and some young women are opting to get married and start families [12]. In many low-resource settings of sub-Saharan Africa, adolescent girls have been seen as a neglected population from previous crises such as the Ebola outbreaks of West Africa, to the current COVID-19 pandemic, resulting in increased maternal mortality and morbidity from teenage pregnancies, and high rates of school drop-out, and turning to informal and potentially risky trades for subsistence [13].

**Figure 1:**
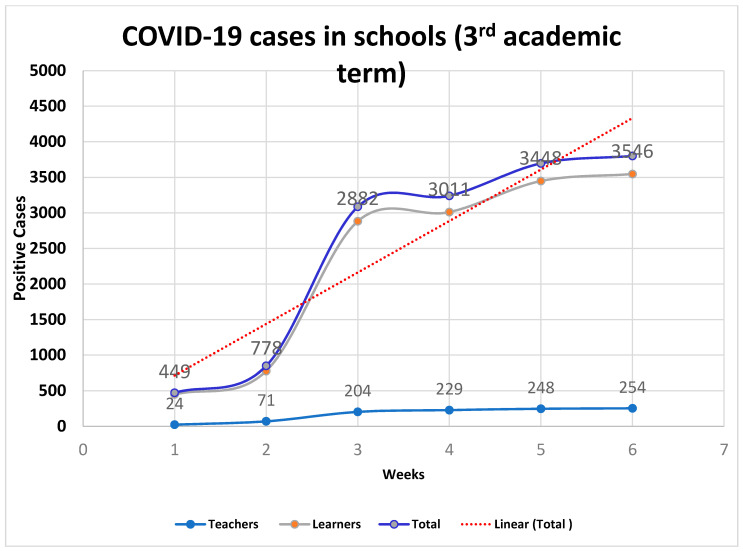
COVID-19 cases in Zimbabwean schools during the third academic term. Source: Ministry of Health and Child Care [14].

When Zimbabwe re-opened schools in September 2021, significant outbreaks of COVID-19 in clusters began to be reported in schools [14], raising the fear that this could degenerate into widespread community transmission, precipitating another wave if not appropriately managed. According to the daily situation reports released by the Ministry of Health and Child Care (MoHCC), there were occasions when more than 50% of the daily cases were attributable to outbreaks in schools [14]. As shown in Figure 1, while the rapid response teams in Zimbabwe moved in swiftly to avert widespread community transmission from clustered cases over the two months of September and October 2021, the contribution of cases linked to schools to the overall burden has remained significant. At this point, the country, like many others, had rolled out vaccination programmes for its adult population, starting from 18 years of age. However, this vaccination programme still excluded all children below 18 years of age, who constitute the bulk of school-going children and make for a significant proportion of the population. We discuss why it is essential to control SARS-CoV-2 transmission within schools in Zimbabwe, discuss vaccination as the most crucial strategy for control, and highlight other important infection prevention and control strategies.

## 2. The Risk of SARS-CoV-2 Infection among Children

Traditional seasonal respiratory viral pathogens tend to affect children more than adults. It was therefore surprising that cases of COVID-19 remained significantly low in the paediatric age groups globally during the early days of the COVID-19 pandemic. With the emergence of mutant variants of concern, especially the delta variant, the risk of contracting SARS-CoV-2, developing symptomatic infection, and hospitalisation increased among school-going children in the United States of America [15]. In Zimbabwe, such cases remained low during the third wave, which occurred between June 2021 and August 2021. This is the harshest epidemic wave experienced by Zimbabwe to date and saw an increase in cumulative confirmed cases from 38,000 to approximately 120,000 [16]. The relative sparing of the children has resulted in arguments and controversies regarding continued school closures amidst increased reports of social ills in this population due to prolonged lockdowns [7]. Evidence of the spread of SARS-CoV-2 in schools [17], including transmission between students and from students to teachers [18], has started to emerge, pointing towards the need to optimise COVID-19 control and mitigation measures in the school going populations. Given that protracted school closures negatively impact children’s educational, physical, and social development, the new evidence gives rise to the need to comprehensively consider the balance between the ills arising from these prolonged closures and the risk of SARS-CoV-2 transmission among the students, their teachers and other service providers. Hence, the need to optimise strategies to make the school environment safer.

Others have argued that schools should only be re-opened when the incidence and prevalence of COVID-19 at the community level has been put under control and once good school-based mitigation strategies have been put in place [19]. Given that the transmission of COVID-19 in the population is occurring in epidemic waves, waiting for total community transmission control would mean repeated disruptions to children’s academic activities when a significant amount of time has already been spent out of school. While children appear to be spared mainly of the direct impacts of COVID-19, the biggest concern is their potential for transmitting SARS-CoV-2 to vulnerable teachers, other workers in schools, and the elderly or vulnerable family members at home in the community. Such transmissions can generate devastating community transmission. With the majority of Zimbabweans not fully vaccinated [20,21], preventing such outbreaks is essential given the fragility and lack of capacity and resilience of the Zimbabwe public health sector to deal with large scale outbreaks.

Zimbabwe has not carried out epidemiological studies to stratify school-going children at risk of COVID-19 infection, and the daily situation reports produced by the MoHCC do not provide such granular data. This would be important for formulating critical COVID-19 control policies in schools, including vaccination. However, age-disaggregated data from the World Health Organization from 30 December 2019 to 25 October 2021 reveals that all child age groups can be infected, with under-fives representing 2% of global cases, those between 5 and 14 years 7% and older adolescents and young adults, 15–24 years representing about 15% of all global cases [22]. Fortunately, deaths for all ages less than 25 years represented less than 0.5% of reported global deaths. Several risk factors for severe COVID-19 in children have been reported, including childhood obesity and pre-existing conditions including diabetes mellitus, asthma, heart and pulmonary diseases, neurologic, neurodevelopmental, and neuromuscular conditions [23]. Children with such conditions must be prioritised for preventive interventions and control strategies. However, it must be noted that these results are from studies from high income countries, and low-income countries such as Zimbabwe need to conduct their own studies in this regard.

As of the third wave of COVID-19 in Zimbabwe which ended by August 2021, the delta variant was responsible for 98% of all the cases in Zimbabwe [16]. Genomic surveillance is a challenge, as the country relies on international collaborations with the Quadram institute for these assays [24]. There is a lack of epidemiological studies to profile the symptomatology of the infection according to age groups in the country; however, again basing on WHO reports and studies from elsewhere, younger children tend to present with milder symptoms of the disease compared to older populations, and some infected children may not exhibit any symptoms at all [22]. With the recently sequenced Omicron variant, which has been detected in samples in neighbouring Botswana and South Africa, it will be important to establish the patterns of disease in children to more appropriately inform control programs.

## 3. COVID-19 Testing among Children in Zimbabwe

COVID-19 testing is essential in schools as it helps to identify, treat, and isolate confirmed cases promptly, and quarantine those who may have been exposed [25]. Surveillance teams can effectively contact-trace all cases and avert widespread community transmission by tracing, for day scholars, those cases with origins back in the community. Hence, testing school children can potentially provide critical information regarding SARS-CoV-2 transmission at the community level, and this information can be used to formulate preventive transmission strategies. The testing in schools has been happening at various levels, with some schools demanding that pupils be tested using rapid antigen tests and bring COVID-19 free certificates before they were admitted into schools while other schools provided testing facilities at the school gates to ensure that only pupils who were COVID-19 free were allowed into the premises [26]. Although these measures have been taken, they also have some challenges as some parents cannot afford to pay for the testing costs; hence some schools are opening without testing pupils. This is due to the high price associated with COVID-19 testing, which results in parents prioritising other school needs, such as paying school fees and buying uniforms and consumables for the pupils.

On the other hand, due to limited resources, the government cannot provide free testing for all the children opening schools except in cases of suspected outbreaks, as has happened at some schools. In instances where schools have suspected cases among pupils, the MoHCC is notified and proceeds to do rapid antigen tests confirmed by SARS-CoV-2 PCR if positive. This has been done several times; for instance, 18 learners who exhibited symptoms and their contacts were tested at one school and nine of them tested positive [26]. In another incident, a boarding school was closed after more than 100 pupils had tested positive for COVID-19 [27].

The Centers for Disease Control (CDC) also recommends that frequent testing, at least once a week, be done for students, teachers and other staff members not fully vaccinated who are involved in high-risk sports and extracurricular activities which are done indoors. Screening testing is also recommended if the school is not tracking COVID-19 vaccination status of participants and support teachers and staff. However, as mentioned above, the costs associated with testing hamper this. Some sectors argue whether testing asymptomatic children has a beneficial public health effect as it may subject young children to unnecessary discomfort and requires parental/guardian consent. The issue of consent also depends on the parents/guardians’ values, goals, and preferences. Therefore, it is necessary to introduce and optimise vaccination of school-going children, the teachers, and other service providers in schools.

## 4. Optimising Vaccination as a Key Strategy for Control of COVID-19 among School-Going Children in Zimbabwe

Zimbabwe’s COVID-19 responses are guided by an intersectoral framework updated periodically through coordinated efforts led by the MoHCC. The broad objective of this framework is to enable high-quality vaccination services and reduce morbidity and mortality due to COVID-19 disease in Zimbabwe. The specific goals include vaccinating the eligible population voluntarily for free up to a minimum of 60% of the total population as the herd immunity threshold (HIT), starting with high-risk target populations. The other objectives include putting in place adequate logistical measures to ensure smooth vaccine supply chains to the recipients, creating sufficient demand for vaccines, ensure the availability of a functional cold chain at all levels, and putting in place a clear strategic information framework to identify strengths, opportunities, weaknesses, and threats of the program.

Vaccination is established as the best preventive public health intervention to deal with the spread, morbidity, and mortality associated with infectious diseases [28]. Since the advent of vaccination programmes for infectious diseases globally, millions of lives have been saved from infectious globally, and some have been eradicated or are near eradicated. Therefore, the approval of SARS-CoV-2 vaccines for use in the general population has been perceived as a major milestone in controlling the COVID-19 pandemic [29]. Several vaccines, including messenger ribonucleic acid (mRNA), adenovirus-vector based and inactivated whole virus vaccines are now available on the market. Unfortunately, from the outset of the programme, only those aged 18 years and above were eligible for SARS-CoV-2 vaccination, and children and other key populations, including pregnant and breastfeeding women, were excluded. Given that these populations constitute a sizeable proportion of the people in many countries, excluding them from vaccination programmes makes it difficult to attain herd immunity thresholds. Gradually, however, countries are starting to develop guidelines for vaccination in these populations.

Official statistics show that as of 8 November 2021, 6,033,470 individuals have received at least one COVID-19 vaccine in Zimbabwe [14]. This is slightly more than 40% of the country’s total population, one of Africa’s highest vaccination rates [30]. Approximately 18% of Zimbabweans are fully vaccinated [14]. The primary vaccines in use in Zimbabwe are Sinovac and Sinopharm, and the Sinovac vaccines have been noted to be 87.5% protective against hospitalisation and 86% against death among fully vaccinated adults [31]. Unfortunately, studies of these vaccines were conducted outside Zimbabwe, and their efficacy against emerging variants of concern remains unknown. Detailed analysis of current vaccination data from Zimbabwe indicates that the more affluent individuals in the country’s two largest cities, Harare and Bulawayo, have received a more significant percentage of vaccines than the rural areas where nearly 70% of the population reside [14].

Until recently, Zimbabwe’s national COVID-19 control strategy was silent about vaccination in young children, despite being quite a comprehensive strategy. As a welcome development, on 2 November 2021, the government circulated a communication approving the vaccination of school-going children between 16 and 17 years with the Sinovac vaccines [32]. Robust evidence for the safety and effectiveness of vaccines among younger children remains scarce, and critical questions regarding this aspect remain a potent driver of vaccine hesitancy. Historically, measles vaccination in the USA suffered a major setback when concerns regarding its association with childhood autism were raised. However, other countries have extended SARS-CoV-2 vaccination to younger children or are running clinical trials to answer vaccine safety questions in this population [33]. Hopefully, this evidence will be established soon to allow vaccination of school-going children and propel countries towards their herd immunity thresholds.

While the decision to expand vaccination to 16–17-year-old age group in Zimbabwe is a welcome development, the announcement should probably have included ages lower than 16 years old, given that other countries are now vaccinating even younger children. Vaccinating the 16–17 year age groups is aimed at protecting a larger proportion of the population, with the aim of close to 100% coverage for this population, and hence propel the country towards its herd immunity threshold. Unfortunately, this currently excludes children with higher risk profiles as discussed above, but who fall below these age ranges, and denies them of the beneficial protection conferred by the vaccines. China, the supplier of the Zimbabwe’s most commonly utilised vaccines, has extended its vaccination program to children aged 3–11 years to increase its mass vaccination [34], and hopefully this can provide answers to some critical questions. The argument of contention remains the unavailability of safety and effectiveness data of the vaccines available for paediatric populations. The lack of this evidence, alongside the dearth of clear communication strategies from the risk communication and community engagement (RCCE) pillar of the MoHCC might drive vaccine hesitancy in this population, as they have done in older people. Zimbabwe’s COVID-19 vaccination programme, like that of other countries, has been suffering from marked vaccine hesitancy and lack of clear information and communication strategies, along with several falsehoods, myths, and misconceptions circulating widely on diverse social media have been noted to be key drivers of this hesitancy [35]. It remains essential for Zimbabwe to identify key drivers of vaccine hesitancy in the concerned population and address them sufficiently to optimise vaccine uptake. Additionally, barriers to access must be overcome as a key and urgent priority, and this involves taking the vaccines right to where the children live or learn to reduce logistical challenges to access. The equitable distribution of vaccines to marginalised children remains as crucial for children as for the adult population. It is the government’s responsibility to ensure proportionate distribution with a bottom-up approach.

Identification of populations where uptake of health promotion and protection is reduced is important, guided by past experiences. In Zimbabwe, it is therefore critical to consider the apostolic sect which has theological objections to vaccination. This may be more so as the origins of COVID-19 and the objectives of its vaccination programmes have been perceived as being associated with evil in some religious sects, and prominent religious leaders in the country have loudly spoken against COVID-19 vaccination. These rumours, falsehoods, myths, and misconceptions have had extensive reach owing to the easy accessibility of diverse forms of social media. Though the Zimbabwe Expanded Programme of Immunisation (ZEPI) is one of the most comprehensive and successful in Africa, uptake of childhood vaccines in this population remains challenging. It, therefore, is imperative that any strategies to facilitate uptake of COVID-19 vaccines must strategically look at how they can optimise uptake in this crucial sect of the population. For the country to reach its target of 60% of its population to have received an a COVID-19 vaccine by the end of December 2021, there is imperative need to favour effective interventions to facilitate uptake, which could be achieved by more rapid vaccine uptake in the 16–17-year-old group and other younger age groups.

While considering an extension of vaccination to schoolchildren is extremely important, it is critical that teachers and other workers in schools be prioritised for vaccination. It has been highlighted that whilst children are relatively spared of devastating morbidity and mortality from COVID-19, their potential to act as vectors of transmission to more vulnerable populations, among which could be teachers, parents, or other vulnerable members in society, remains a significant public health concern. For this reason, clear, non-coercive strategies to optimise teacher vaccination are critical. Alongside this, there is clear need for the RCCE pillar of the MoHCC to find ways of reaching these childrens’ guardians, especially in the marginalised rural and farming areas of Zimbabwe where poor road networks and transport challenges often serve as significant barriers to reach, resulting in suboptimal uptake of public health programmes.

Instilling confidence in vaccines to improve their uptake requires comprehensive strategies that consider the whole population. Guardians of the school-going children include women of reproductive age, an important group with special concerns, as they consider fertility intentions and the safety of their foetuses and children seriously before taking up vaccines [36]. Unless the MoHCC comes up with guidelines for vaccination in this population, which are currently not available [21], uptake of vaccines among the children of this group might remain problematic. Therefore, it is imperative that the MoHCC expedite the development of vaccination guidelines for this key population, alongside clear communication and health promotion strategies to improve uptake.

## 5. Other Infection Prevention and Control Strategies Remain Vital in Schools

Following WHO and CDC guidance, adhering to the other infection prevention and control strategies for SARS-CoV-2 remains vital in schools as it is in communities. With the country far from attaining its herd immunity threshold and school children generally unvaccinated for now, these remain the cruxes of control. This is also important because the duration of immunity conferred by COVID-19 vaccines remains unestablished now, and evidence suggests reduced efficacy against emerging variants of concern for some of the vaccines. Countries with reduced capacity for regular genomic surveillance, such as Zimbabwe, may not always be aware of the circulating variants. Insights from genomic surveillance are helpful in informing public health programmes, including vaccination.

While it is challenging to promote physical distancing in schools, especially among the younger children, who cannot endure facemasks for prolonged periods, serious attempts must be made, especially by the more senior grades. To this end, the educators collaborating with the RCCE pillar in the MoHCC need to develop innovative ways appealing to young children for communicating issues relating to COVID-19. Colourful, age-appropriate information, education and communication (IEC) material illustrating such aspects as physical distancing, hand-sanitising, and hand-washing is needed and must be developed urgently. Many schools have resorted to making children wear face shields, but there is no evidence suggesting that they have a preventive role in the school environment and may only make children uncomfortable.

Other settings have developed health check applications that can assess the risk of COVID-19 daily [37]. Such apps input data, including symptoms and travel history, more beneficial than daily temperature checks. Schools must develop screening algorithms to detect children who may have active COVID-19 to be tested, treated, and isolated appropriately. Though daily temperature checks have been widely adopted in schools, there is no good evidence to support their use as a screening tool. To avoid exporting SARS-CoV-2 infections from schools into communities, boarding schools need facilities that allow isolation and monitoring of infected students within the schools, with appropriate tracing and quarantining of their contacts within the same areas. Unless there are significant large scale outbreaks, it is prudent that schools remain open. The CDC recommends that everyone wear a mask that covers the mouth and nose, washes frequently, and set a goal for the physical distancing of six feet [38]. Since it is difficult to consistently enforce physical distance among students, especially the elementary ones, there is a need to ensure adequate ventilation by opening windows and doors and thoroughly cleaning the surfaces.

## 6. Conclusions

Notwithstanding that Zimbabwe has made great strides in making vaccines readily available for its citizenry, children have been left out. Efforts must be urgently made using efficacy and pharmacovigilance data from countries that have made vaccines available to children to approve the provision of vaccines for this group, which constitute the largest segment of the population and a pivotal contributor to infection.

Although the vaccine supply of Sinopharm and Sinovac has been to a great extent reliable and for Zimbabwe to rapidly achieve herd immunity, it must increase its usage of the COVAX facility to improve its vaccine supply chain, especially when children become eligible to receive vaccinations. The country will need to import at least an additional 10 million doses in a short period. Innovative strategies to overcome theological rigidity and cultural barriers existing in the apostolic groups to uptake COVID-19 testing and vaccination must be prioritised to enable widespread uptake in this population.

All stakeholders responsible should develop Information, Education, and Communication (IEC) materials tailored towards the school-going population according to age and cultural groups. The IEC materials should include infographics, flyers, and leaflets in different formats, including social media. Additionally, contact tracing and isolation in the case of boarding schools may be necessary. Finally, schools should not be closed to provide a measured and efficient response to the threats posed by the COVID-19 epidemic. The use of multiple prevention strategies reported here has shown that when outbreaks in school settings are adequately managed, the transmission usually is low. Still, rather infection prevention and control strategies, including vaccination and surveillance in schools, heightened. This, alongside their positive impact on the health, development, and wellbeing of children, would justify that they are maintained open when possible.

## Data Availability

Not applicable.
